# Visuomotor Prediction Errors Modulate EEG Activity Over Parietal Cortex

**DOI:** 10.1038/s41598-018-30609-0

**Published:** 2018-08-21

**Authors:** F.-A. Savoie, F. Thénault, K. Whittingstall, P.-M. Bernier

**Affiliations:** 1 Département de médecine nucléaire et radiobiologie, Faculté de médecine et des sciences de la santé, Université de Sherbrooke, Sherbrooke, Quebec Canada; 2 Centre de Recherche du CHUS, Université de Sherbrooke, Sherbrooke, Quebec Canada; 3 Département de kinanthropologie, Faculté des sciences de l’activité physique, Université de Sherbrooke, Sherbrooke, Quebec Canada

## Abstract

The parietal cortex is thought to be involved in visuomotor adaptation, yet it remains unclear whether it is specifically modulated by visuomotor prediction errors (*i.e*. PEs; mismatch between the predicted and actual visual consequences of the movement). One reason for this is that PEs tend to be associated with task errors, as well as changes in motor output and visual input, making them difficult to isolate. Here this issue is addressed using electroencephalography. A strategy (STR) condition, in which participants were instructed on how to counter a 45° visuomotor rotation, was compared to a condition in which participants had adapted to the rotation (POST). Both conditions were matched for task errors and movement kinematics, with the only difference being the presence of PEs in STR. Results revealed strong parietal modulations in current source density and low theta (2–4 Hz) power shortly after movement onset in STR *vs*. POST, followed by increased alpha/low beta (8–18 Hz) power during much of the post-movement period. Given recent evidence showing that feedforward and feedback information is respectively carried by theta and alpha/beta oscillations, the observed power modulations may reflect the bottom-up propagation of PEs and the top-down revision of predictions.

## Introduction

Current motor control theories propose that the brain uses inverse and forward internal models to generate motor commands and predict their sensory consequences^1–3^. To ensure optimal accuracy of motor behaviour across development and ageing, the relationship between motor output and sensory input must be continuously calibrated^1,2^. This process, known as sensorimotor adaptation, is thought to be driven by the mismatch between the predicted and actual sensory consequences of movement (*i.e*. prediction errors, PE)^4^.

It is well documented that the cerebellum contributes to the processing of PEs that lead to sensorimotor adaptation^5–12^. For instance, recent work in non-human primates has provided strong evidence for a PE signal arising from neurons within the rostral fastigial nucleus when voluntary movements are perturbed by a resistive force^5^. Furthermore, several studies have shown that insult to the cerebellum disrupts adaptation in both humans^6,8,10,13–16^ and monkeys^17^. Yet, in spite of considerable research, the contribution of neocortical brain regions to the processing of sensorimotor PEs remains unclear. In the context of visuomotor adaptation, a reasonable hypothesis is that regions lying along the dorsal visual stream, namely the parietal cortex, could take part in this process. Indeed, the parietal cortex receives direct visual projections^18–20^ and is involved in anticipatory limb-state estimation through forward modeling^3,21,22^, making it functionally suited to encode visuomotor PEs. In support, neuroimaging work has often reported increased parietal activity upon initial exposure to a visuomotor perturbation, a finding compatible with the representation of a PE^7,23–27^. Moreover, lesions to the posterior parietal cortex have been shown to impede visuomotor adaptation^28,29^. Finally, diffusion imaging in humans^30^ and axonal tracing work in non-human primates^31,32^ have provided evidence for structural connectivity between the intraparietal sulcus and the cerebellum, raising the possibility that these regions interact for the processing of visuomotor PEs^33^. In this light, the main goal of the present investigation was to assess whether visuomotor PEs modulate visually evoked responses over parietal scalp sites using electroencephalography (EEG).

One challenge in investigating the neural basis of visuomotor PEs is that when initially exposed to a visuomotor perturbation, participants often miss the intended target (*i.e*. they make task errors), conflating neural activity associated with the processing of visuomotor PEs with that associated with the processing of reward PEs (*i.e*. the mismatch between the predicted and actual result of an action^34^). This is a concern, given that adaptation is known to occur despite null task errors^4,35–38^, which implies that these error signals are at least partially independent. Another limitation is that visuomotor perturbations alter visual feedback and result in iterative changes in motor output whenever participants repeatedly face the perturbation, making it difficult to determine whether differences in brain activity reflect a visuomotor PE or low-level sensory- or motor-related changes.

To circumvent these limitations, the present study compared EEG activity between a strategy condition, in which participants were provided with a strategy to counter a visuomotor rotation, and a post-adaptation condition, in which participants had adapted to the rotation. Previous work has shown that when using a strategy, participants implicitly adapt to the perturbation despite null task errors^4,35–38^, indicating that visuomotor PEs are experienced. In post-adaptation trials, visuomotor PEs are assumed to be reduced since repeated exposure to a visuomotor rotation results in the update of an internal model^4,39,40^. Critically, task errors, motor output and visual input would be matched between strategy and post-adaptation trials, such that contrasting the EEG data from both conditions would allow to isolate visuomotor PE-related neuronal dynamics.

## Methods

### Participants

Fifteen healthy male university students (mean age 25 ± 4 yrs, range 21–35 yrs) with normal or corrected-to-normal vision participated in this EEG study. Given that 1) this study did not focus on a priori determined EEG frequency bands and 2) there is a paucity of data concerning parietal oscillatory responses to visuomotor PEs, the sample size of the present study was not determined with an a priori power analysis. Instead, sample size was based on that of previous studies that investigated sensorimotor processes using EEG (Arrighi *et al*.^41^, N = 12; Perfetti *et al*.^42^ N = 17; Torrecillos *et al*.^43^, N = 15; Torrecillos *et al*.^44^, N = 15, N = 14 and N = 10; Tan *et al*.^45^, N = 12; Tan *et al*.^46^ N = 17). All participants were right-handed based on self-report, as well as the Edinburgh Handedness Inventory^47^. The experimental procedures were approved by the institutional review board and ethics committee of the Université de Sherbrooke and performed in accordance with the guidelines and regulations imposed by this committee. All participants gave their informed written consent prior to participation.

### Experimental set-up

Participants were seated at a desk, facing a vertically-oriented CRT monitor (Studioworks 995E, LG, Seoul, South Korea). A digitizing tablet (DrawingBoard VI, CalComp, Scottsdale, Arizona, USA), which was used to control a cursor on the monitor (green circle; radius 0.3 cm), was placed on the desk horizontally immediately in front of participants and centered with respect to the monitor. Participants controlled the cursor by displacing a stylus on the surface of the digitizing tablet with their right hand. Throughout the experiment, a custom-made box covered the digitizing tablet, such that participants could not see their arm or hand, but freely move the stylus across the tablet’s surface.

### Experimental task

**Figure 1:**
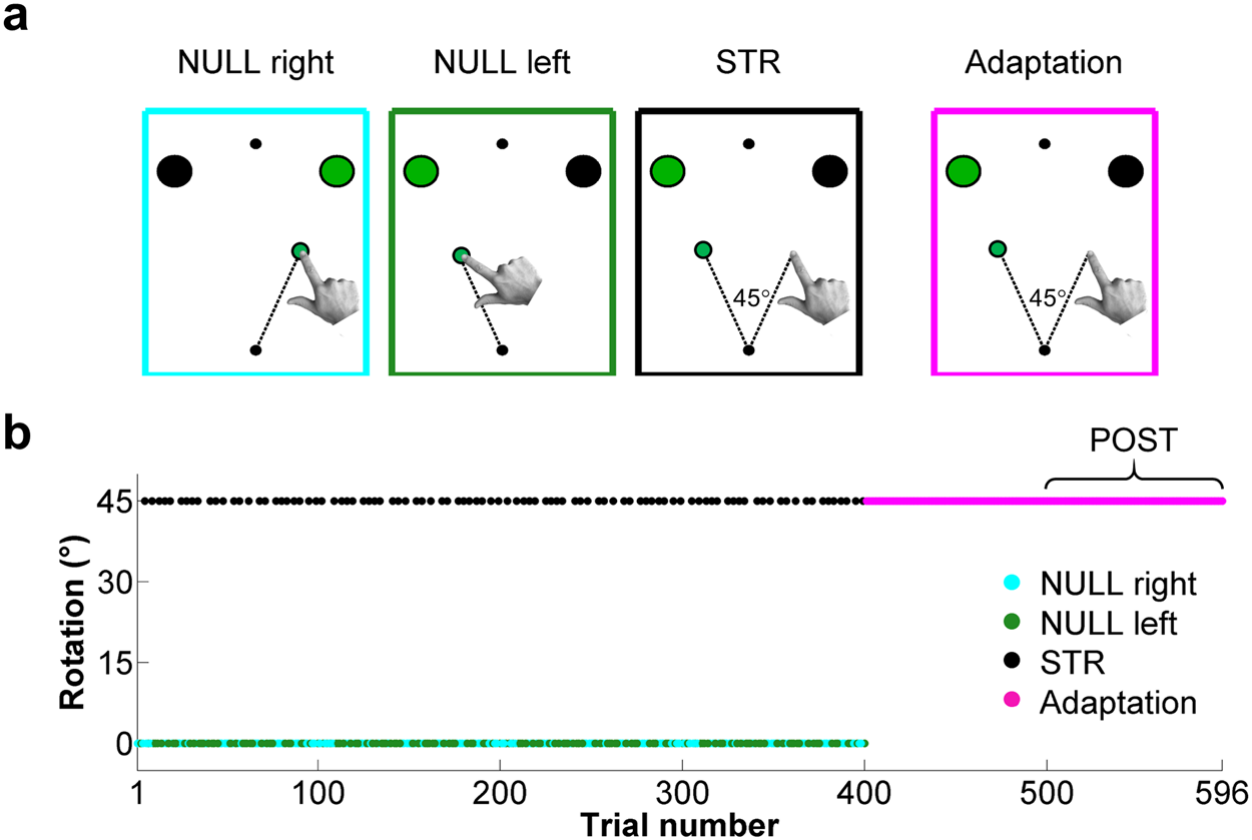
Experimental conditions and perturbation schedule. (**a**) Visual representation of the hand and cursor displacement during NULL right (cyan), NULL left (green), STR (black) and adaptation (magenta) trials. (**b**) Angle of visuomotor rotation throughout the experiment. During the first 400 trials, each pair of STR trials was interspersed by at least 2 NULL (right or left) trials. Visuomotor rotation was continuously present during the final 196 trials, the last 96 of which were binned as the post-adaptation (POST) condition.

The experimental paradigm consisted of a center-out reaching task to one of two visual targets (Fig. 1a). All reaches were initiated from a start circle (white; radius 0.5 cm), which was centered with respect to participants’ midline. The two targets (white circles; radii: 0.7 cm) were located 8 cm away from the start circle and positioned 22.5° on either side of the midline. A fixation point (white circle; radius 0.5 cm) was positioned 8 cm away from, and directly above, the start circle. Participants were instructed to fixate this circle throughout the experiment to prevent eye movements. All visual landmarks (*i.e*. start circle, targets and fixation point) were present on the monitor at all times.

Trials were initiated when participants moved the cursor within the start circle. When this occurred, one of the two targets turned red (pre-cue), indicating that this was the target to acquire on the ensuing reach. Simultaneously, an auditory tone was delivered to inform participants of which experimental condition they would face during that trial. After a 2500 ms delay, the target turned green, prompting the initiation of the reach (*i.e*. go-cue). Participants were instructed to “strike through” the cued target by executing straight ballistic movements lasting ~300 ms. During both the experiment and familiarization period (see Experimental protocol and conditions), whenever movement times exceeded 350 ms for three consecutive trials, participants were instructed to increase their movement speed during the following intertrial interval. This ensured that movement times were consistent during the experiment. Visual feedback of the cursor was provided within an 8 cm radius around the start circle, beyond which it was extinguished. Participants were instructed to terminate their movements 1–2 cm past the target location and were instructed to hold their final hand position for 2000 ms, after which the cursor reappeared and signalled the end of the trial.

### Experimental protocol and conditions

The experiment consisted of five blocks of 100 trials and a sixth block of 96 trials. During the first 400 trials, participants were faced with 3 possible types of trials: Null rotation reaches to either the right (NULL right) or left (NULL left) targets (152 trials each, Fig. 1a first and second panels) or strategy (STR) reaches to the left target (96 trials, Fig. 1a third panel). In the NULL right and NULL left trials, the position of the visual cursor precisely reflected that of the hand on the digitizing tablet. In contrast, in the STR trials, the position of the visual cursor was rotated 45° counterclockwise with respect to the position of the hand. As mentioned above, at the onset of all trials (*i.e*. when participants moved the cursor into the start circle), an auditory tone was delivered to inform participants of the type of condition they would face during that trial. For NULL right and NULL left trials, the auditory cue was a low pitch (100 Hz) tone, whereas in the STR condition, the auditory cue was a high pitch (300 Hz) tone. Prior to the experiment, participants were informed that these tones indicated whether a strategy should be used to avoid missing the intended target due to visuomotor rotation. Specifically, participants were told: “*When you hear the high-pitch auditory tone at trial onset, the cursor will be rotated 45° counterclockwise with respect to hand position. Therefore, to successfully bring the visual cursor to the left target, you must “strike through” the target to the right*”. The purpose of the NULL right and NULL left trials was to washout any adaptation that could occur as a result of the STR trials and thus maximize the magnitude of visuomotor PEs on every STR trial. Hence, the first 400 trials were pseudorandomized so that at least two NULL (left or right) trials separated any pair of STR trials (Fig. 1b).

It is believed that adaptation results in the update of the predicted sensory consequences of movement to better predict subsequent movement-related re-afferences^48–51^, thereby minimizing the cost of cortical responses^52^ and optimizing motor control^53–55^. Hence as adaptation takes place, visuomotor predictions should gradually better account for the perturbed visual input, thereby reducing visuomotor PE magnitude. As such, trials recorded after extended exposure to a visuomotor rotation should result in visuomotor PE attenuation. Hence during the fifth and sixth blocks of the experiment (196 trials), participants underwent an adaptation phase during which they repeatedly had to bring the visual cursor to the left target under 45° counterclockwise visuomotor rotation (Fig. 1b). These trials were thus in every way identical to those of the STR condition, except that they were not interspersed with null rotation trials. Bond and Taylor^56^ have demonstrated that implicit adaptation accounts for ~25° of the angular compensation participants make after 80 reaches made to a single target under 45° visuomotor rotation, with additional movements having only a minor impact on implicit adaptation. Given that implicit adaptation is believed to reflect the updating of a forward model^40^, it was reasoned that 100 adaptation trials would induce sufficient visuomotor remapping to attenuate visuomotor PEs compared to STR trials. Therefore, the final 96 adaptation trials were pooled together and defined as the post-adaptation (POST) condition, against which STR trials would be compared to isolate neural activity related to visuomotor PE. To ensure that participants fully understood the task and were at ease with producing movements lasting ~300 ms, they were submitted to at least one practice block, consisting of 50 null rotation trials interleaved with 10 STR trials, prior to data collection.

### Kinematic data recording and analysis

Visual stimuli were presented using functions from the psychophysics toolbox (Psychtoolbox^57,58^), which were run with MATLAB (v2014a, MathWorks, Natick, MA, USA) using the Windows 7 operating system (Microsoft, Redmond, WA, USA) on a desktop computer (Dell Optiplex 7010, Round Rock, Texas, USA). All hand position-related data, obtained from the digitizing tablet, were recorded at 100 Hz and analyzed offline with custom MATLAB routines. Movement initiation and termination were respectively defined as the moments when the stylus moved outside the start circle and when stylus velocity became ≤1.5 pixel/s after its radial distance from the start base exceeded 8 cm. For each trial, the following variables were determined. Reaction time (RT) was calculated as the difference between the go-cue and movement initiation. Movement time (MT) was calculated as the difference between movement initiation and termination. Peak velocity was determined by differentiating the *x* and *y* stylus positions as a function of time and identifying the largest resultant velocity during MT. Time to peak velocity was identified as the moment, from movement initiation, when peak velocity was attained. Hand *vs*. visual target angle at peak velocity was also determined, taken as the angular difference between the visual target vector (*i.e*. start circle to visual target position) and hand vector (*i.e*. start circle to hand position) at peak velocity. Final hand position, defined as the absolute distance between the locations of the hand at movement termination and the aiming target was determined for each trial.

### Kinematic-based trial rejection

To help ensure kinematic homogeneity across conditions, trials for which RT or MT were ≤150 ms or ≥600 ms were discarded. Moreover, to ensure that differences in task errors (*i.e*. reward PEs) did not differ across conditions, only trials in which the cursor hit the intended target were retained for analysis. This was done by rejecting trials for which the pixels representing the target and cursor failed to overlap at any point during the reach (*i.e*. target miss). All 596 experimental trials were submitted to this trial rejection process. In STR, 3 ± 4, 2 ± 3 and 11 ± 6 trials were respectively found not to meet the RT, MT and target hit criteria, whereas in POST, this equated to 4 ± 4 (RT), 3 ± 4 (MT) and 10 ± 6 (target hits) trials. Thus overall, 13 ± 6 and 14 ± 6 trials were rejected in STR and POST, respectively.

### EEG data acquisition, processing and time-frequency decomposition

Scalp EEG was recorded using a 64-electrode actiCAP (Brain Products, Gilching, Germany) and BrainAmp system (Brain Products, Gilching, Germany). The electrodes were positioned according to the extended 10/20 system and Cz placed over the vertex of participants’ head during recording. EEG data were digitized online and sampled at 500 Hz using the BrainVision Recorder software version 2.0 (Brain Products, Gilching, Germany), using a laptop (Dell Latitude E6530, Round Rock, Texas, USA) running on Windows 7 (Microsoft, Redmond, WA, USA). All EEG analyses were done offline using custom MATLAB routines, as well as functions derived from the EEGLAB^59^ and CSD^60^ toolboxes. EEG signals were bandpass filtered between 1–100 Hz, with a 59–61 Hz notch to eliminate landline artifacts and re-referenced to the average scalp potential. The data were then epoched from −2500 ms to +2500 ms around movement onset. EEG data were then baseline-corrected to the average potential recorded during the 500 ms preceding movement onset. This period was chosen as a baseline because it immediately preceded the visuomotor PE, which was expected to occur when movement-related visual re-afference would be available (*i.e*. during the movement). Once baseline corrected, trials that had been rejected based on movement kinematics were discarded from the EEG datasets. After this, single-trial data were screened for non-stereotypical EEG artifacts. Specifically, trials that showed scalp potential deflections >150 µV, which corresponded to 4 ± 5 trials for STR and 4 ± 3 trials for POST, were excluded from analyses. Thus, analyses (both EEG and movement kinematics) were conducted on a total trial count of 79 ± 9 and 79 ± 6 for STR and POST, respectively. The data were then submitted to independent component analysis (ICA), a blind separation technique that decomposes the EEG signal into maximally independent components^61^, using the “runica” algorithm from the EEGLAB toolbox. ICA is a standard method for removing artifacts from EEG activity without having to discard entire epochs^61,62^. A component was identified as being artifactual if it met two of the following three criteria: (1) its power spectrum was not generally found to decrease with frequency, as EEG and EMG spectral power are respectively expected to decrease^63^ and increase^64^ as a function of frequency; (2) its topography showed sources at the far edges of the scalp, as these components often reflect eye movement and muscle artifacts^61^ and (3) its time-course showed spurious bursts of activity. The remaining “clean” components were back-projected to electrode space. Finally, the data were transformed into current source density (CSD) activity using the Surface Laplacian transform (*m-*constant, 4; head radius, 10 cm; smoothing constant, 10^−5^)^60^. The decision to transform EEG data into CSD activity was motivated by evidence showing that it improves the spatio-temporal resolution of EEG dynamics compared to monopolar recordings^65,66^. Moreover, it has been shown that transforming scalp potentials to CSD following ICA artifact rejection is an efficient way to remove undesired electromyographic activity from the EEG signal^67^, since the Surface Laplacian diminishes the sensitivity of the recording sites to distant sources^68^.

In addition to CSD activity, time-frequency analyses were carried out to better characterize the neural responses to visuomotor PEs. This was done by convolving the EEG time series of each electrode and trial with a series of complex Morlet wavelets spanning 1–100 Hz in 1 Hz intervals. The cycle count for the wavelets was linearly increased from 3–12.9 in 0.1 steps to limit frequency smoothing at higher frequencies^69^. An estimate of power was obtained for all frequencies and time points, by squaring the amplitude of the complex signal resulting from convolution. Thereafter, median power values were determined for each electrode, time point and frequency. The median was used as the central tendency measure for the single participant event-related spectral perturbation (ERSP) data because in contrast with the arithmetic mean, the median is only trivially affected by outlier data^69^. The obtained median power time-series were then baseline-normalized by way of a decibel conversion using the following equation:

1 $${\rm{dB}}=10\ast log10\,({\rm{RP}}/\overline{{\rm{BP}}})$$

where dB corresponds to the decibel-converted median power, RP the median power value at a given time point, and $\overline{{\rm{BP}}}$ the average raw power during the baseline period (−500 to 0 ms) obtained from the median power time-series. It should be noted that all baseline corrections (CSD) and normalizations (ERSPs) were made on a *per* condition basis. This rules out the possibility that differences in EEG responses across conditions are attributable to changes in baseline brain activity over time.

### EEG data analysis

The objective of the present study was to investigate whether visuomotor PEs are associated with differential EEG activity at parietal scalp sites. Since the Surface Laplacian acts as a spatial filter that increases the contribution of radial electric potentials to scalp recordings^69^, the neural generators of the recorded CSD activity are assumed to lie in close vicinity (*i.e*. 2–3 cm^65^) of the recording electrodes. Therefore, the EEG data were examined using a right parietal region of interest (ROI) comprising electrodes P2, P4 and PO4, as these electrodes have been shown to lie directly over the superior parietal lobule in neuroimaging and modeling studies^70–72^. The ROI was placed over the right hemisphere because the magnitude of visually evoked potentials is known to be greater at electrodes contralateral to the hemifield in which visual feedback is presented^73^. Hence, given that visual feedback of the cursor was presented to the left of gaze during both STR and POST (Fig. 1a, panels 3 and 4), it was reasoned that visuomotor PE-related EEG modulations would be greatest in the right hemisphere.

In addition to parietal areas, recent evidence suggests that activity within frontal brain regions could also index visuomotor PEs. For instance, there is evidence for the involvement of the prefrontal^74–76^ and anterior cingulate^9,75^ cortex during visuomotor adaptation. Furthermore, EEG studies have shown that a waveform akin to the feedback-related negativity (FRN), a negative potential thought to originate from the anterior cingulate cortex^77^, is present following reach errors caused by a force field^43^, as well as a visuomotor rotation^78^. For this reason, the EEG data from a mid-frontal ROI, consisting of electrodes Fz, FC1 and FC2, was also analysed. These electrodes were chosen because they are known to best capture the FRN response^34,79,80^.

The data recorded from the electrodes within each ROI were pooled together to yield CSD time-courses for each condition, which were submitted to statistical analyses. For spectral power analyses, frequency bands showing the most prominent ERSPs were selected from a condition-averaged spectrogram for each ROI, that is, a spectrogram resulting from the average ERSPs during STR and POST. This ensured that the selection of frequency bands for analyses was orthogonal to the ensuing statistical tests (*i.e*. there was no “double-dipping”^81^).

### Statistical analyses

To assess whether they were normally distributed prior to statistical analyses, all dependent variables (RT, MT, peak velocity, time to peak velocity, hand *vs*. visual target angle at peak velocity, final hand position and EEG data) were submitted to a Shapiro-Wilk test using IBM SPSS Statistics (IBM, North Castle, New York, USA). Given that (1) several of these variables were found to be non-normally distributed (*i.e*. RT, MT and much of the EEG data), (2) the sample size was relatively small (n = 15) and (3) statistical contrasts would largely be limited to paired comparisons between STR and POST, Wilcoxon signed rank tests were used to compare conditions using MATLAB (v2014a, MathWorks, Natick, MA, USA). All statistical tests were two-tailed and the threshold for significance was set to 0.05.

Statistical analyses on CSD time courses were computed as follows: First, the data points comprised between 100 and 400 ms were averaged into 15 contiguous 20-ms time bins. Wilcoxon signed rank tests were then applied to these time-bins to determine whether CSD activity differed between STR and POST. It was decided to only compare CSD activity 100 to 400 ms after movement onset because event-related potentials associated with visual processing, as well as the FRN, typically occur within this time range^73,79,82^. Additional “peak to peak” analyses were also performed. Specifically, the visual N1 component, a negative scalp potential ascribed to exogenous visual stimulus responses^73,82^, and the FRN^34,79,80^ were investigated in this manner. Visual N1 amplitude was calculated as the difference between the peak CSD negativity recorded at the right parietal ROI between 125–200 ms and the average CSD activity 0–50 ms after movement onset, since a clear P1 component could not be identified for all participants^82^. The FRN was computed as the difference between the most positive and negative CSD activity peaks recorded at the mid-frontal ROI 200–600 ms after movement onset^80^. For the ERSP data, statistical analyses were conducted by parsing the individual data points into 50 contiguous 20 ms time bins (0 to 1000 ms). Wilcoxon signed rank tests were then applied to all 50 time bins to determine whether the STR and POST time-courses showed statistically significant differences.

To counter the inflation of type 1 error rate due to multiple comparisons in the analysis of both CSD and ERSP time-courses, *p* values were corrected using the Benjamini-Hochberg, or false discovery rate (FDR), procedure^83,84^. Briefly, the FDR procedure consists of two steps. First, the statistical *p* values are ranked in decreasing order. Second, the *p* values are iteratively (*i.e*. from largest to smallest) compared with a significance threshold adjusted for the FDR, which is obtained from the following equation:

2 $$\mathrm{FDR}{\rm{\alpha }}={\rm{\alpha }}\ast (({\rm{k}}+1-{\rm{n}})/{\rm{k}})$$

where FDRα is the adjusted significance threshold, α is the established significance threshold (*i.e*. alpha, 0.05), k is the number of comparisons and n is the rank of the *p* value. The highest ranked *p* value found to be ≤FDRα is deemed statistically significant, as well as all lower ranked *p* values. For the CSD and ERSP analyses, the number of comparisons (k) was equal to the number of time bins used for analysis, which amounted to 15 (CSD) and 50 (ERSPs). For all analyses, the signed rank statistic (the sum of the ranks of positive differences between STR and POST, W), z-statistic (z), statistical significance (FDR-corrected *p* values) and effect size (Pearson’s r^85^) are reported in the text. According to Cohen^86^, the thresholds past which Pearson’s r denotes small, moderate and large effect sizes are 0.1, 0.3 and 0.5, respectively.

As mentioned above, pre-defined ROIs (*i.e*. right parietal and mid-frontal) were used to test specific spatial hypotheses. An inherent limit to this approach, however, is that it precludes an appreciation of differences in activity that could occur outside of the ROIs. Thus, to provide a broader depiction of modulations in neural activity, scalp maps representing the z-scored Wilcoxon signed rank statistics at each channel were produced for the time windows showing significant differences at each ROI.

## Results

### Kinematic results

**Figure 2:**
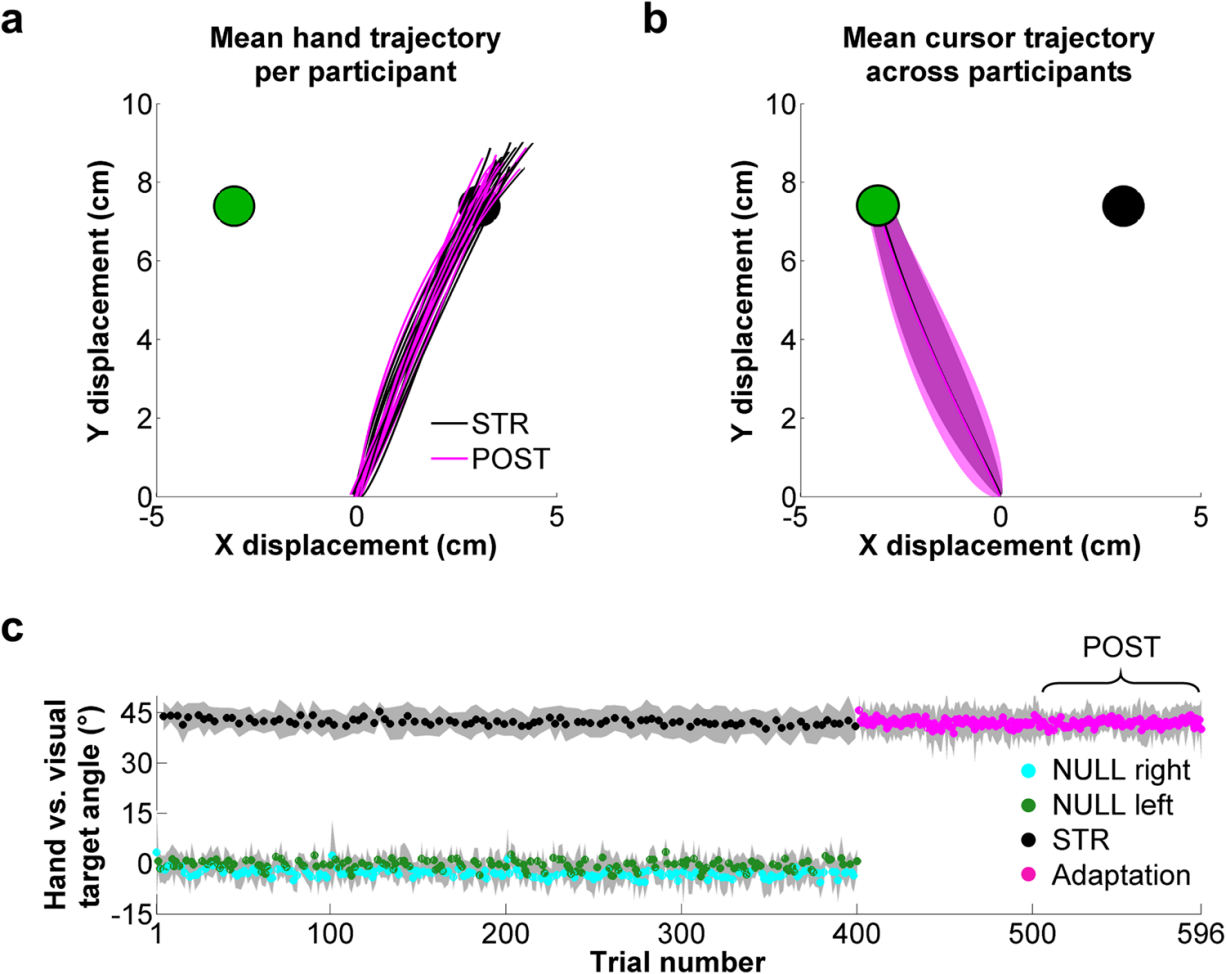
Reach trajectories and hand *vs*. visual target angle at peak velocity. (**a**) Mean hand trajectory for each participant and (**b**) mean cursor trajectory across participants during STR (black) and POST (magenta). (**c**) Hand *vs*. visual target angle at peak velocity throughout the experiment for NULL right (cyan), NULL left (green), STR (black) and adaptation (magenta) trials.

As can be seen in Fig. 2a,b, hand and cursor trajectories were highly similar between STR and POST. Moreover, there were no statistically significant difference between STR and POST for RTs (STR, 362 ± 40 ms; POST, 371 ± 38 ms, *W* = 34, z = −1.5, *p* = 0.14, r = 0.27), MTs (STR, 299 ± 41 ms; POST, 312 ± 81 ms, *W* = 60, z = 0, *p* = 1, r = 0), peak velocity (STR, 60 ± 8 cm/s; POST, 57 ± 12 cm/s, *W* = 84, z = 1.4, *p* = 0.17, r = 0.25), time to peak velocity (STR, 93 ± 17 ms; POST, 97 ± 25 ms, *W* = 44, z = −0.9, *p* = 0.36, r = 0.16) or hand *vs*. visual target angle at peak velocity (STR: 42 ± 3°, POST: 42 ± 3°, *W* = 68, z = 0.5, *p* = 0.65, r = 0.09, Fig. 2c). The only movement-related variable that differed significantly between STR and POST was final hand position. Specifically, final hand position was slightly but significantly farther from the target in STR than in POST, both on the x-axis (STR: 0.87 ± 0.29 cm, POST: 0.64 ± 0.29 cm, *W* = 102, z = 2.4, *p* = 0.017, r = 0.44) and the y-axis (STR: 1.5 ± 0.29 cm, POST: 1.28 ± 0.24 cm, *W* = 103, z = 2.4, *p* = 0.015, r = 0.44).

If visuomotor PEs were experienced during STR trials, hand direction would be expected to drift in the direction opposite to the rotation (i.e. clockwise drift) on the following trial due to implicit adaptation^4,35–38^. Hence to test whether visuomotor PEs were indeed experienced in STR, hand *vs*. visual target angle at peak velocity was compared for trials immediately preceding (STR^−1^) and following (STR^+1^) STR trials. Akin to all other statistical analyses, Wilcoxon signed ranked tests were used to compare the data obtained from STR^−1^ and STR^+1^ trials directed to the left (i.e. NULL left) and right (i.e. NULL right) targets.

For NULL left trials, this analysis revealed a significant difference in hand *vs*. visual target angle at peak velocity between STR^−1^ and STR^+1^ trials (STR^−1^, −0.37 ± 2.28°; STR^+1^, 0.80 ± 2.29°, *W* = 119, z = 3.35, *p* = 0.0008, r = 0.61), with STR^+1^ trials showing clockwise hand drift in 14 out of the 15 participants. For NULL right trials, a similar pattern was observed in 11 of the 15 participants, though the difference between STR^−1^ (−3.19 ± 2.87°) and STR^+1^ (−2.80 ± 3.23°) was not statistically significant (*W* = 89, z = 1.64, *p* = 0.10, r = 0.30). One explanation for this non-significant finding is that adaptation is known to decrease as a function of distance from the target direction^87^, reaching near-zero asymptote for targets ±60° away from the training target. Hence because the right target was positioned +45° from the visual target goal in STR (i.e. the left target), lesser adaptation would be expected for NULL right, compared to NULL left, trials following STR. In any case, the clockwise hand drift observed in STR^+1^ *vs*. STR^−1^ trials in the NULL left condition suggests that the imposed visuomotor rotation resulted in implicit adaptation and thus visuomotor PEs.

### Right Parietal ROI

**Figure 3:**
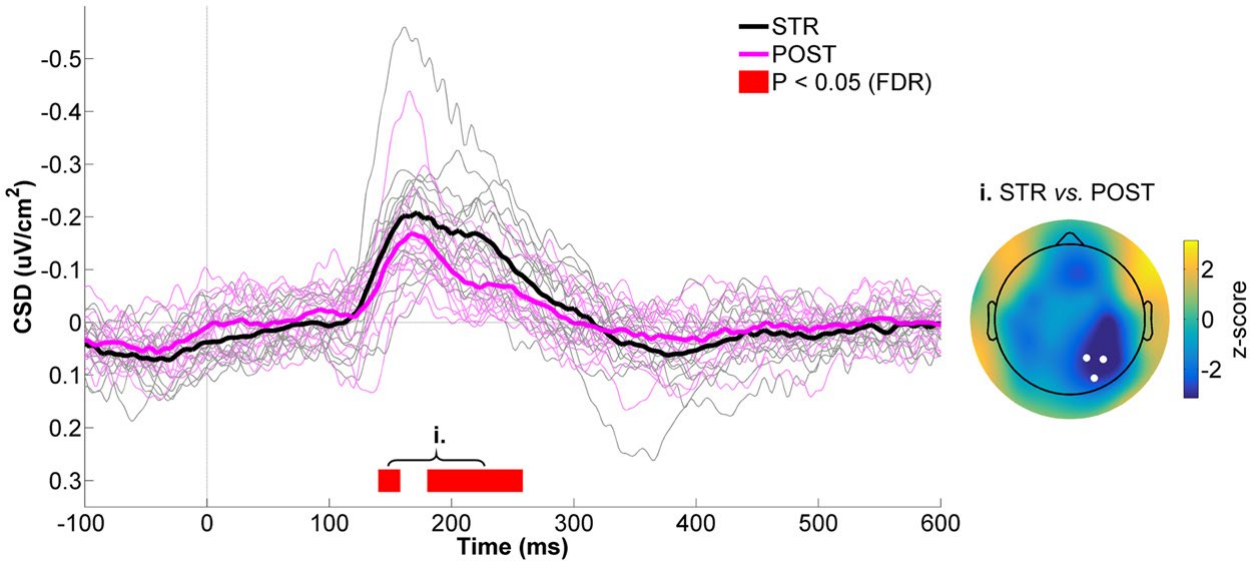
CSD activity recorded at the right parietal ROI for STR *vs*. POST. The black and magenta waveforms respectively represent CSD activity for STR and POST. The thin waveforms represent individual participant data, whereas the thick waveforms represent the mean CSD activity across participants. The red line highlights the time bins showing statistically significant differences between STR and POST (*p* ≤ 0.05, FDR-corrected). Time “0 ms” corresponds to movement onset. The scalp map represents the statistical z-scores across electrodes for CSD activity between STR and POST during the significant time window (i. 140–260 ms). The electrodes that make up the right parietal ROI (P2, P4 and PO4) are highlighted in the scalp map.

Figure 3 presents the CSD time-courses of STR and POST for the right parietal ROI. As can be seen in this figure, a typical visual N1 component was observed in both STR and POST ~170 ms after movement onset, indicative of visual stimulus processing^73,82^. At the moment of, and for a short period after the N1, CSD activity differences started to emerge across conditions. Specifically, CSD values were more negative in STR than POST (140–160 ms: *W* = 11, z = −2.8, *p* = 0.0018, r = 0.51; 180–260 ms: *W*_range_ = [0, 16], z_range_ = [−3.4, −2.4], *p*_range_ = [0.0002, 0.0042], r_range_ = [0.46, 0.62]), with the peak difference occuring at ~220 ms. Further attesting of these differences, the peak to peak amplitude of the visual N1 component was significantly greater in STR than POST (STR: −0.26 ± 0.13 µV/cm^2^; POST: −0.19 ± 0.11 µV/cm^2^; *W* = 6, z = −3.1, *p* = 0.002, r = 0.57). The scalp map presented in the right panel of Fig. 3 confirms that the STR *vs*. POST statistical differences were maximal at right parietal scalp sites.

**Figure 4:**
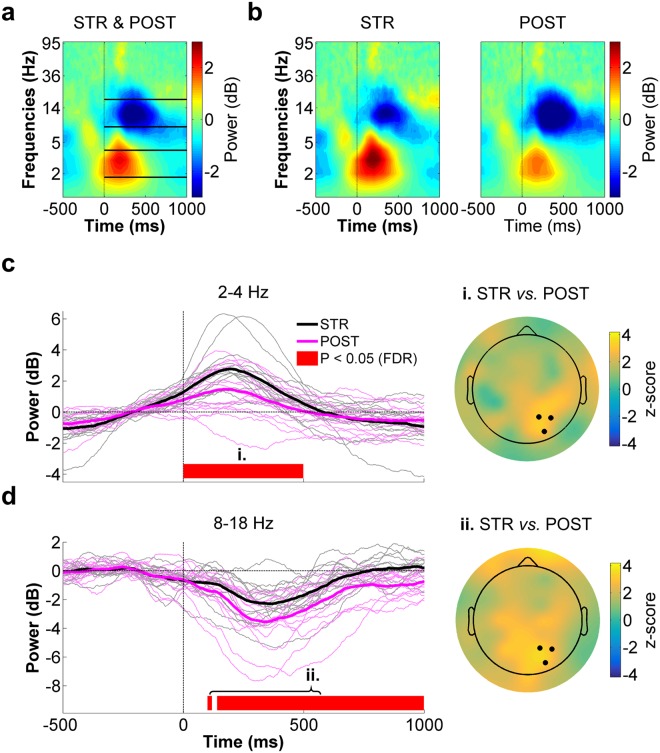
Right parietal ROI time-frequency data for STR *vs*. POST. (**a**) Spectrogram representing the mean ERSPs for STR and POST. The solid lines delimit the frequency bands selected for statistical analyses (*i.e*. 2–4 Hz and 8–18 Hz) Time “0 ms” corresponds to movement onset. (**b**) STR (left panel) and POST (right panel) ERSPs. (**c**) Low theta (2–4 Hz) spectral power time-course for STR (black) and POST (magenta) (left panel). The scalp map (right panel) represents the statistical z-scores across electrodes for low theta power differences between STR and POST during the significant time window (i. 0–500 ms). (**d**) Alpha/low beta (8–18 Hz) spectral power time-course for STR (black) and POST (magenta) (left panel). The scalp map (right panel) represents the statistical z-scores across electrodes for alpha/low beta power differences between STR and POST during the significant time window (ii. 100–1000 ms). In the left panels of both (**c**) and (**d**), the thin waveforms represent individual participant data, the thick waveforms represent the mean power across participants and the red line highlights the time bins showing statistically significant differences between STR and POST (*p* ≤ 0.05, FDR-corrected). In the right panels of both (**c**) and (**d**), the highlighted electrodes denote those that make up the right parietal ROI (P2, P4 and PO4).

The preceding CSD results indicate that cortical activity differed at right parietal electrodes between STR and POST despite identical motor output, sensory input and the absence of task errors. The next analyses sought to determine how visuomotor PEs altered ERSPs at the same electrodes. The spectrogram presented in Fig. 4a corresponds to the average ERSP for STR and POST (respectively shown in Fig. 4b) for the right parietal ROI. As can be seen within the solid lines plotted in this spectrogram, the most important ERSPs were identified in the low theta (~2–4 Hz) and alpha/low beta (~8–18 Hz) frequency bands. These were therefore statistically compared between STR and POST.

The time-course for 2–4 Hz power is presented in Fig. 4c. As can be seen, low theta power increased in both conditions shortly after movement onset, with the increase being more pronounced in STR as compared to POST. This was confirmed by statistical analysis which revealed a significant difference between STR and POST during the first 500 ms after movement onset (*W*_range_ = [100, 119], z_range_ = [2.3, 3.4], *p*_range_ = [0.0004, 0.012], r_range_ = [0.41, 0.61]). The z-score scalp map presented in the right panel of Fig. 4c clearly shows that low theta power differences between STR and POST were maximal at right parietal scalp sites.

The time-course of alpha/low beta (8–18 Hz) power, is depicted in Fig. 4d. Within this frequency range, a transient desynchronization was observed in both STR and POST, followed by a synchronization. Overall, alpha/low beta power was greater during STR than POST, with significant differences for most of the time bins comprised between 100 to 1000 ms (*W*_range_ = [96, 120], z_range_ = [2.0, 3.4], *p*_range_ = [0.0006, 0.036], r_range_ = [0.37, 0.62]). The spatial distribution of this difference, which spanned a large bilateral and caudal area including the right parietal ROI, can be appreciated in the scalp map shown in the right panel of Fig. 4d.

Although the findings presented above suggest that right parietal areas are sensitive to visuomotor PEs, the reported differences could be attributable to the fact that POST trials were collected consecutively and at the end of the experiment, when participants may have started feeling fatigued. Consequently, the EEG differences between STR and POST may have been caused by (1) repetition suppression, which occurs with repeated exposure to the same visual stimulus^88,89^; (2) fatigue-related changes in brain state or (3) participants paying less attention or neglecting the redundant visual feedback in POST. To rule out these possibilities, right parietal responses were compared between STR and NULL left, as these conditions were matched for visual feedback and were recorded in an alternated manner during the same experimental blocks (see Methods). Importantly, because NULL left trials were recorded with veridical visual feedback before adaptation took place, it was reasoned that like POST, the magnitude of the visuomotor PE experienced during NULL left would be lesser than in STR. Hence if the STR *vs*. NULL left contrast results in EEG differences similar to those observed in the STR *vs*. POST contrast, it can reasonably be posited that the differences result from visuomotor PE processing.

**Figure 5:**
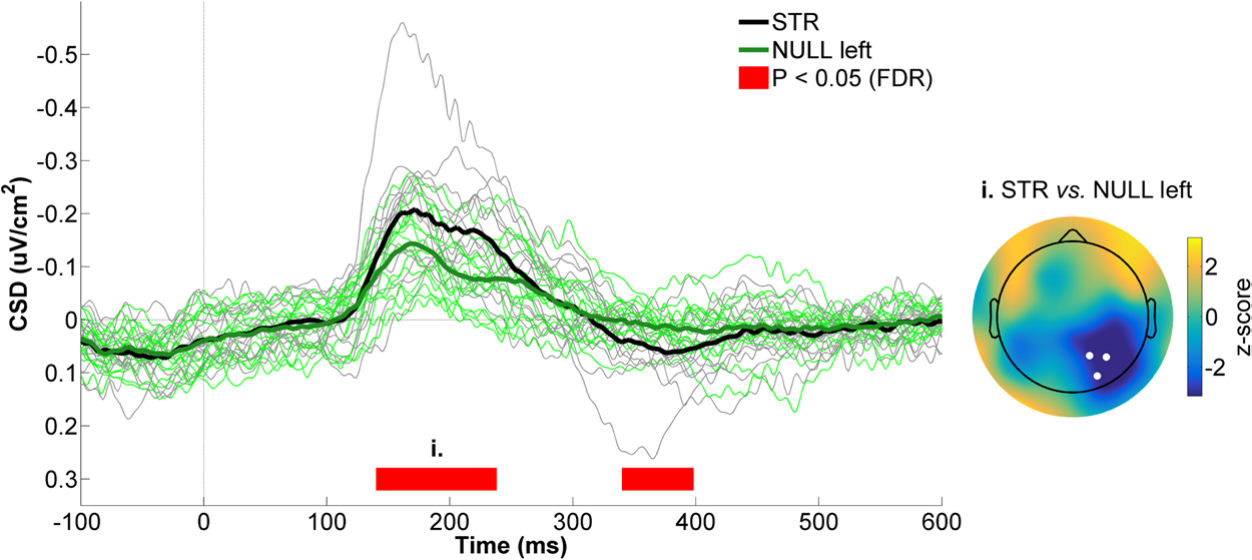
CSD activity recorded at the right parietal ROI for STR *vs*. NULL left. The black and green waveforms respectively represent CSD activity for STR and NULL left. The thin waveforms represent individual participant data, whereas the thick waveforms represent the mean CSD activity across participants. The red line highlights the time bins showing statistically significant differences between STR and NULL left (*p* ≤ 0.05, FDR-corrected). Time “0 ms” corresponds to movement onset. The scalp map represents the statistical z-scores across electrodes for CSD activity between STR and NULL left during the significant time window (i., 140–240 ms). The electrodes that make up the right parietal ROI (P2, P4 and PO4) are highlighted in the scalp map.

**Figure 6:**
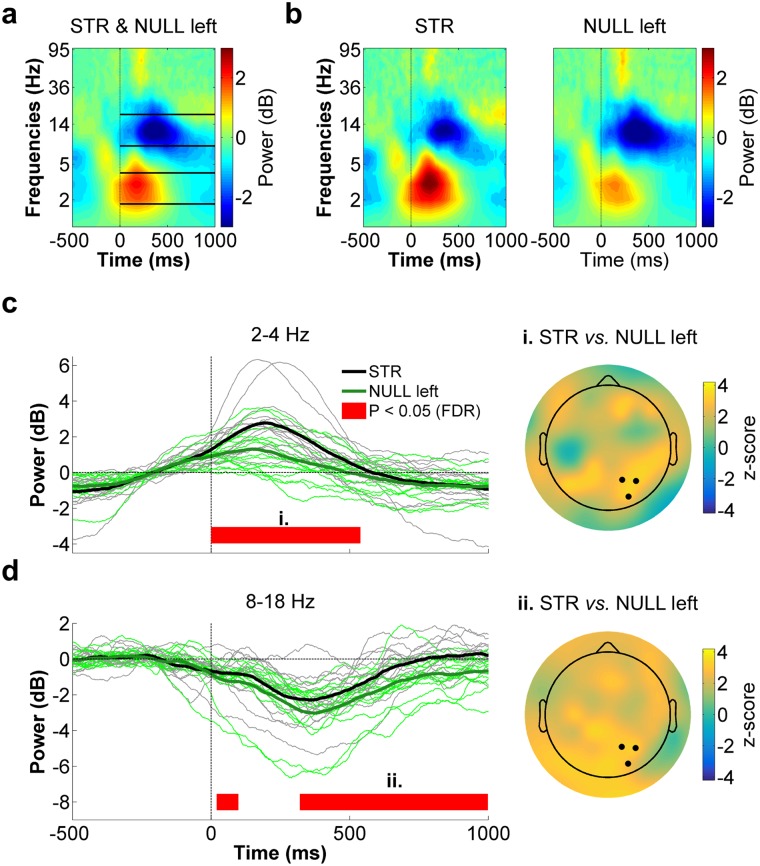
Right parietal ROI time-frequency data for STR *vs*. NULL left. (**a**) Spectrogram representing the mean ERSPs for STR and NULL left. The solid lines delimit the frequency bands selected for statistical analyses (*i.e*. 2–4 Hz and 8–18 Hz). Time “0 ms” corresponds to movement onset. (**b**) STR (left panel) and NULL left (right panel) ERSPs. (**c**) Low theta (2–4 Hz) spectral power time-course for STR (black) and NULL left (green) (left panel). The scalp map (right panel) represents the statistical z-scores across electrodes for low theta power differences between STR and NULL left during the significant time window (i. 0–520 ms). (**d**) Alpha/low beta (8–18 Hz) spectral power time-course for STR (black) and NULL left (green) (left panel). The scalp map (right panel) represents the statistical z-scores across electrodes for alpha/low beta power differences between STR and NULL left during the significant time window (ii. 360–1000 ms). In the left panels of both (**c**) and (**d**), the thin waveforms represent individual participant data, the thick waveforms represent the mean power across participants and the red line highlights the time bins showing statistically significant differences between STR and NULL left (*p* ≤ 0.05, FDR-corrected). In the right panels of both (**c**) and (**d**), the highlighted electrodes denote those that make up the right parietal ROI (P2, P4 and PO4).

The following statistical analyses were conducted exactly as was done for the STR *vs*. POST contrast (see Statistical analyses). As can be seen in Fig. 5, CSD values were more negative in STR than in NULL left from 140 to 240 ms (W_range_ = [0, 20], z_range_ = [−3.4, −2.3], *p*_range_ = [0.0003, 0.012], r_range_ = [0.41, 0.62]), whereas the opposite was true from 340 to 400 ms (W_range_ = [102, 115], z_range_ = [2.38, 3.12], *p*_range_ = [0.001, 0.009], r_range_ = [0.44, 0.57]). Similar to the differences observed in the STR *vs*. POST contrast, early (140 to 240 ms) differences between STR and NULL left were maximal over right parietal scalp sites (Fig. 5, right panel). With respect to ERSPs (Fig. 6a,b), low theta (2–4 Hz) spectral power was significantly greater during STR than NULL left from 0 to 520 ms (W_range_ = [100, 120], z_range_ = [2.3, 3.4], *p*_range_ = [0.0003, 0.012], r_range_ = [0.41, 0.62], Fig. 6c). In the alpha/beta frequency range, spectral power was greater in STR than NULL left from 20 to 100 ms (W_range_ = [97, 101], z_range_ = [2.10, 2.32], *p*_range_ = [0.015, 0.027], r_range_ = [0.38, 0.43]) as well as from 360 to 1000 ms (W_range_ = [98, 118], z_range_ = [2.16, 3.3], *p*_range_ = [0.0008, 0.024], r_range_ = [0.39, 0.60], Fig. 6d). Hence overall, EEG differences between STR and NULL left were quite similar to those observed between STR and POST, which argues against the possibility that the differences observed between the latter conditions resulted from the late and consecutive recording of POST trials.

### Mid-Frontal ROI

**Figure 7:**
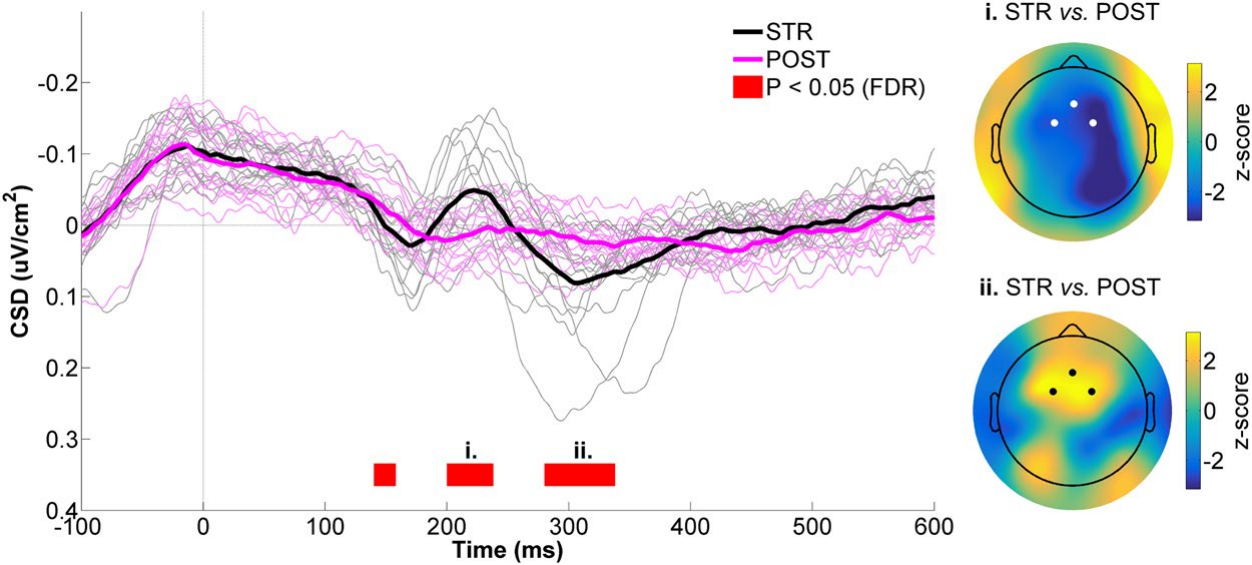
CSD activity recorded at the mid-frontal ROI for STR *vs*. POST. The black and magenta waveforms respectively represent CSD activity for STR and POST. The thin waveforms represent individual participant data, whereas the thick waveforms represent the mean CSD activity across participants. The red line highlights the time bins showing statistically significant differences between STR and POST (*p* ≤ 0.05, FDR-corrected). Time “0 ms” corresponds to movement onset. The scalp maps represent the statistical z-scores across electrodes for CSD activity between STR and POST during two significant time windows (i. 200–240 ms; ii. 280–340 ms). The electrodes that make up the mid-frontal ROI (Fz, FC1 and FC2) are highlighted in both scalp maps.

At the mid-frontal ROI (Fig. 7, left panel), an early difference in CSD activity was observed between STR and POST during the 140–160 ms time bin (W = 112, z = 3.0, *p* = 0.0013, r = 0.54). Soon after, CSD activity showed a strong negative deflection in STR, while no meaningful CSD modulation occured in POST. This led to a significant difference between conditions, with STR showing greater negativity as compared to POST (200–240 ms: *W*_range_ = [5, 6], z_range_ = [−3.1, 3.0], *p*_range_ = [0.0007, 0.0009], r_range_ = [0.56, 0,57]). The spatial distribution of this STR *vs*. POST difference can be appreciated in the upper right panel of Fig. 7. As can be seen, strong differences were observed at mid-frontal electrodes, as well as at right parietal scalp sites, consistent with the data presented in the right panel of Fig. 3. It should also be noted here that the mid-frontal difference between STR and POST resembled a classic FRN, with the peak negativity occuring ~220 ms after feedback provision^34,79^. Peak to peak analyses confirmed this finding, as the amplitude of this FRN-like potential was significantly greater in STR (−0.19 ± 0.10 µV/cm^2^) than POST (−0.11 ± 0.03 µV/cm^2^, *W* = 4, z = −3.2, *p* = 0.001, r = 0.58). Finally, CSD activity in STR markedly increased after the FRN-like waveform, becoming significantly more positive than in CTRL between 280 and 340 ms (*W*_range_ = [101, 112], z_range_ = [2.3, 3.0], *p*_range_ = [0.0013, 0.008], r_range_ = [0.42, 0.54]). The z-score scalp map for this time window is provided in the lower right panel of Fig. 7, and confirms a clear positive CSD difference at mid-frontal scalp sites.

**Figure 8:**
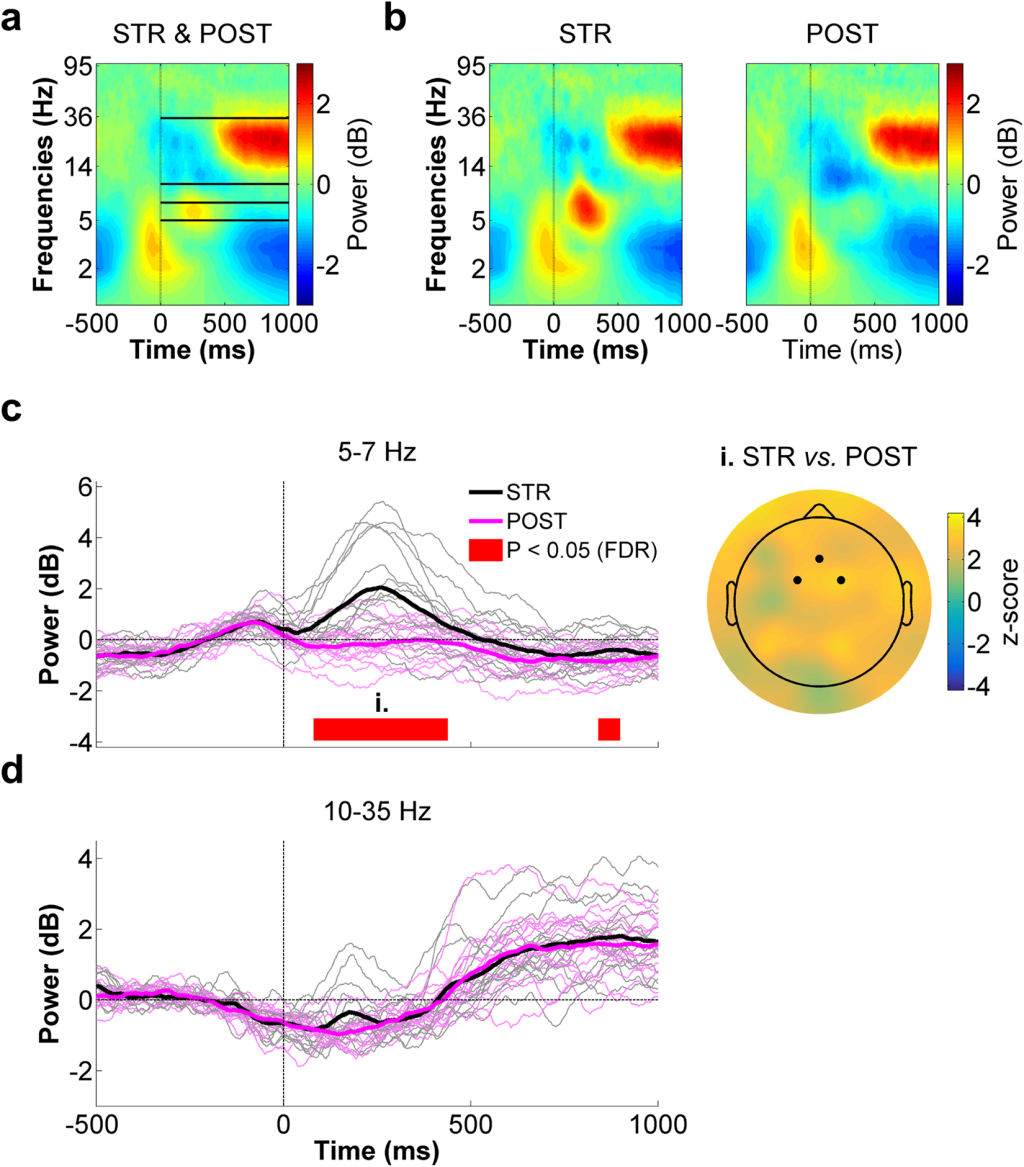
Mid-frontal ROI time-frequency data for STR *vs*. POST. (**a**) Spectrogram representing the mean ERSPs for STR and POST. The solid lines delimit the frequency bands selected for statistical analyses (*i.e*. 5–7 Hz and 10–35 Hz) Time “0 ms” corresponds to movement onset. (**b**) STR (left panel) and POST (right panel) ERSPs. (**c**) High theta (5–7 Hz) spectral power time-course for STR (black) and POST (magenta) (left panel). The red line highlights the time bins showing statistically significant differences between STR and POST (*p* ≤ 0.05, FDR-corrected). The scalp map (right panel) represents the statistical z-scores across electrodes for high theta power differences between STR and POST during the significant time window (i., 80–440 ms). The highlighted electrodes denote those that make up the mid-frontal ROI (Fz, FC1 and FC2). (**d**) Beta (10–35 Hz) spectral power time-course for STR (black) and POST (magenta). In the left panels of both (**c**) and (**d**), the thin waveforms represent individual participant data, whereas the thick waveforms represent the mean power across participants.

Figure 8a presents the average spectrogram for STR and POST (respectively shown in the middle and right panels of Fig. 8b) at the mid-frontal ROI. As can be seen, clear ERSPs could be identified in the high theta (~5–7 Hz) frequency range shortly after movement onset, as well as in a wide beta (~10–35 Hz) frequency band. Accordingly, statistical analyses between STR and POST were carried out on 5–7 Hz and 10–35 Hz spectral power. As can be seen in the time-course depicted in Fig. 8c, high theta power showed a marked increase soon after movement onset in STR, whereas it slightly decreased during this time period in POST. This led to a significant difference across conditions between 80–440 ms (*W*_range_ = [96, 120], z_range_ = [2.3, 3.2], *p*_range_ = [0.0005, 0.008], r_range_ = [0.42, 0.59]). A significant difference was also identified between 840–900 ms (*W*_range_ = [102, 108], z_range_ = [2.4, 2.7], *p*_range_ = [0.0027, 0.0072], r_range_ = [0.44, 0.50]), with STR showing greater spectral power than POST. A z-score scalp map of the early 5–7 Hz power difference between STR and POST is presented in the right panel of Fig. 8c. As can be seen, mid-frontal channels showed strong differences between conditions. However, similarly high z-scores were found at several other scalp sites, indicative of widespread neural activity differences in this frequency band.

With respect to the 10–35 Hz frequency band, spectral power density was found to desynchronize during the movement period, afterwhich it synchronized in both STR and POST (Fig. 8d). Although the time bins spanning 140–220 ms appeared to show differences within this frequency range, these were not found to be statistically significant after FDR correction (*W*_range_ = [100, 108], z_range_ = [2.3, 2.7], *p*_range_ = [0.22, 0.32], r_range_ = [0.41, 0.50]). Overall, no statistically significant differences were identified within the 10–35 Hz range (*W*_range_ = [36, 108], z_range_ = [−1.4, 2.7], *p*_range_ = [0.22, 1.08], r_range_ = [0, 0.50]).

## Discussion

The extent to which sensorimotor PEs are distributed across multiple brain areas and the distinguishing functional roles of these areas is an important issue in sensorimotor neuroscience. The goal of the present study was to investigate whether visuomotor PEs modulate visually evoked responses over parietal brain regions, while controlling for task errors, motor output and visual input. The main finding is that visuomotor PEs incurred potent modulations in both CSD and oscillatory activity at parietal electrodes contralateral to the visual reafferent feedback. This suggests that areas lying along the dorsal visual stream, such as the parietal cortex, are likely involved in the processing of visuomotor PEs.

With respect to CSD activity, visuomotor PEs manifested as increased negativity over parietal electrodes contralateral to the visual feedback, with the difference between STR and POST peaking ~220 ms after movement onset. This result finds echo in the literature on action-effect anticipation, which has provided evidence for larger evoked responses when visual stimuli are unpredicted^88,90,91^. For instance, in a motor task similar to the one used here, Benazet *et al*.^82^ showed that imperceptibly delaying hand visual feedback by ~150 ms increased the amplitude of the visual N1 component compared to veridical visual feedback. While being qualitatively similar, the present findings differ in one notable aspect from action-effect anticipation studies. Indeed, participants typically cannot anticipate the deviant stimulus in action-effect paradigms, because they are unaware of when or where it will be presented. This was not the case in the present study, as participants were pre-cued regarding the perturbation at the onset of each STR trial. The fact that they were able to correctly reach toward the opposite target is direct evidence that they anticipated the rotated visual feedback. The current findings therefore extend previous work in that awareness of the perturbation is not enough to prevent a visuomotor PE. This speaks to the low-level nature of the observed error process and suggests that the conscious anticipation of visual feedback is fundamentally different from the sensory predictions that are associated with descending motor commands. In this light, it is tempting to speculate that the observed parietal response is linked to the seminal findings of Mazzoni and Krakauer^4^, who showed that implicit adaptation cannot be overridden by an explicit strategy during visuomotor adaptation.

In the time-frequency domain, visuomotor PEs were associated with an early phasic increase in low theta (2–4 Hz) spectral power at right parietal scalp sites. Given the temporal and spatial similarity of this effect to the preceding CSD modulation, it may have reflected the same phenomenon. Indeed, the low theta power increase most likely corresponded to phase-locked oscillatory power, which is thought to strongly contribute to event-related potentials^69,92^. Slightly after the theta-band modulations, visuomotor PEs were associated with an increase in alpha/low beta (8–18 Hz) power over a broad parietal region, which lasted for much of the post-movement period. Interestingly, this pattern of oscillatory modulations is in good agreement with predictive coding theory, which emphasizes that the brain predicts sensory events to better infer their causes and facilitate perception^52^. A key postulate from this framework is that only the residual signals resulting from discrepancies between bottom up sensory inputs and top down predictions (*i.e*. PEs) propagate up the sensory hierarchy^93,94^. In a seminal study, Arnal *et al*.^95^ used an audiovisual task and investigated oscillatory activity within the superior temporal sulcus, a region at the convergence of visual and auditory inputs, when auditory inputs invalidated visual speech. They found that audiovisual PEs were associated with early correlated modulations in theta- (5–6 Hz) and gamma-band (70–90 Hz) activity, followed by an increase in post-stimulus beta-band (14–15 Hz) activity in the same region. They suggested that PEs are propagated upstream mainly through coupled theta- and gamma-band oscillations, whereas predictions (and their revisions) are transmitted downstream through the beta channel. More directly relevant to the current findings is recent electrocorticography work probing feedforward and feedback signaling within the primate visual system, revealing that feedforward influences are carried by theta- (∼4 Hz) and gamma-band (∼60–80 Hz) synchronization, whereas feedback influences are carried by beta-band (∼14–18 Hz) synchronization^96^. Given this, a possible explanation of the present results is that the early phasic theta-band response reflected the rapid propagation of a PE signal along the dorsal visual stream, whereas the alpha/low-beta-band response reflected the update of the visual predictions, a likely prerequisite to adapt visuomotor transformations.

To identify neural activity pertaining to visuomotor PE processing, the main contrast in this study was between STR and POST. This contrast was chosen because (1) motor output, visual input and task errors were matched between these conditions and (2) following 100 trials of adaptation, visuomotor PEs were hypothesized to be smaller in POST than STR trials^56^. The POST condition, however, differed from the STR conditions in a few respects, notably in that trials were recorded consecutively and collected systematically later in the experiment. Hence, alternative explanations for the EEG differences observed between STR and POST could be that they resulted from repetition suppression^88,89^, fatigue-related changes in brain state or from participants simply neglecting the visual feedback during POST trials. The comparison between STR and NULL left, however, argues against these possibilities. Specifically, the EEG differences between STR and NULL left highly resembled those observed between STR and POST, despite the fact that NULL left trials were embedded amongst STR and NULL right trials (*i.e*. NULL left trials were hardly ever recorded consecutively). Moreover, NULL left trials were collected during the same experimental blocks as STR trials, making fatigue-related differences in brain state unlikely. Another alternative explanation for the present findings is that right parietal EEG differences were due to unmatched visuospatial attention between STR and POST. Indeed, since visuospatial attention is known to increase the magnitude of contralateral visually evoked responses^97^, it could have been that the larger CSD negativity recorded in STR than POST was caused by visuospatial attention being directed more leftward in STR. However, the similar CSD difference identified in the STR *vs*. NULL left contrast also argues against this interpretation, since visuospatial attention was likely directed leftward at least as much in NULL left as in STR. Finally, it could be argued that the observed EEG modulations were caused by the small difference in final hand position between STR and POST. This is unlikely for two reasons. First, visual feedback was not provided past the target, thus equating visual feedback amplitude across conditions. Second, the peak difference in CSD and 2–4 Hz power between STR and POST occurred before movement termination (~220 ms and ~250 ms, respectively). Hence if related, differences in final hand position were arguably the result, rather than the cause, of the observed EEG modulations. Thus overall, the present results point to visuomotor PEs as the most likely cause of the EEG differences observed between STR and POST.

The current view in motor control is that the parietal cortex provides an internal representation of the state of the body in space by integrating incoming sensory feedback with cerebellar predictions derived from an efference copy of descending motor commands^3^. Indeed, there is good evidence that the cerebellum and parietal cortex are respectively involved in predicting the sensory consequences of the movement^98–100^ and representing the current state of the body^21,22,101^. In light of the present results, an important issue relates to the respective contributions of the cerebellum and parietal cortex to the representation of PEs. One possibility, consistent with the observed oscillatory modulations, is that visuomotor PEs are directly computed within the parietal cortex. This would be supported by theoretical and empirical work showing that the laminar organization of the neocortex can support local PE computations through the encoding of residual sensory signals unaccounted for by predictions^52,93,94^. These PE signals may then be communicated to the cerebellum to serve as teaching signals to update the forward model and increase the accuracy of future predictions. An alternative possibility is that PE signals are generated in the cerebellum and are transmitted to neocortical sensorimotor regions, such as the parietal cortex, to modify synaptic weights within the parieto-frontal network during adaptation^33^. This would be consistent with considerable evidence for sensorimotor PE-like activity in the cerebellum^5,12,27^. Still, in light of the often-reported co-activation of parietal and cerebellar regions during adaptation, it may also be that they compute complementary PE-signals, perhaps subserving different aspects of the sensorimotor adaptation process. Future work should test whether the modulations in parietal and cerebellar activity during visuomotor adaptation depend on one another, perhaps through experimental disruption of either brain area using neurostimulation.

Simultaneous with the CSD differences identified at right parietal electrodes, mid-frontal electrodes also showed greater CSD negativity in STR as compared to POST. The timing (~220 ms) and scalp topography of this potential largely resembled the FRN. This is further supported by the fact that a hallmark of the FRN is increased theta power in its time-frequency representation^102,103^, which was also observed. A FRN-like potential has been shown to occur following reach errors caused by a force field^43^, as well as visuomotor rotation^78^. Likewise, Arrighi *et al*.^41^ have recently reported increased mid-frontal theta power following prism-induced reach errors. However, the designs used in these previous investigations made it difficult to determine whether the recorded activity was due to a sensorimotor PE, a reward PE or a combination of both. By controlling for task errors, the present work demonstrates that visuomotor PEs alone do give rise to a FRN-like potential and increased theta power over mid-frontal scalp sites, suggesting that rostral brain areas, possibly the anterior cingulate cortex^77,103^, are indeed sensitive to this type of error. In light of the prevailing view that activity in the medial prefrontal cortex reflects performance-ameliorating processes related to reinforcement learning or strategy implementation^104,105^, it is possible that the present modulations were associated with a more cognitive/conscious evaluation of the visuomotor PE, rather than its implicit representation^104^. In this regard, it has been hypothesized that implicit adaptation processes, presumably mediated by a parieto-cerebellar network^33^, are necessary for the explicit aspects of adaptation to spontaneously develop^37^. Hence, given that implicit adaptation processes were engaged in the STR condition, a reasonable hypothesis is that the frontal EEG modulations resulted from PE signals being communicated to a frontal performance monitoring network, allowing for the implementation of rapid trial-by-trial changes in aiming direction to optimize behaviour. In support, mid-frontal theta oscillations have been proposed as a neural mechanism for organizing performance feedback^79,102,106^. Given the above, the FRN and underlying theta power increase may relate to the fast^107^ or explicit^108,109^ component of adaptation.

One limitation of this work is the lack of behavioral evidence that participants had adapted to the visuomotor rotation by the time POST trials were recorded, as after-effects were not measured following adaptation. One may therefore suspect that adaptation never occurred in this condition, despite that participants were repeatedly exposed to the rotation. This seems unlikely, however, as participants showed a clockwise drift in hand direction following STR trials, which suggests that the visuomotor rotation did elicit implicit adaptation. Future studies should avoid this methodological shortcoming and measure either after-effects or another visuomotor recalibration proxy following adaptation.

In sum, the present study provides evidence that visuomotor PEs modulate visually evoked responses over parietal scalp sites, while controlling for task errors, motor output and visual input. This suggests that neocortical regions actively take part in the processing of PEs, possibly in conjunction with the cerebellum. More work is warranted to elucidate the cellular bases of these error signals and to determine whether they can be exploited to optimize motor rehabilitation.

## Data Availability

The datasets analysed in the present study are available from the corresponding author upon reasonable request.
